# Influence of Folate-Targeted Gold Nanoparticles on Subcellular Localization and Distribution into Lysosomes

**DOI:** 10.3390/pharmaceutics15030864

**Published:** 2023-03-07

**Authors:** Raffaella Daniele, Chiara Brazzale, Busra Arpac, Francesco Tognetti, Cristiano Pesce, Alessio Malfanti, Edward Sayers, Francesca Mastrotto, Arwyn T. Jones, Stefano Salmaso, Paolo Caliceti

**Affiliations:** 1 Department of Pharmaceutical and Pharmacological Sciences, University of Padova, Via F. Marzolo 5, 35131 Padova, Italy; 2 UCLouvain, Louvain Drug Research Institute, Advanced Drug Delivery and Biomaterials, Avenue Mounier 73 B1.73.12, 1200 Brussels, Belgium; 3 Cardiff School of Pharmacy and Pharmaceutical Sciences, Cardiff University, Redwood Building, King Edward VII Ave, Cardiff CF10 3NB, UK

**Keywords:** gold nanoparticles, folate targeting, intracellular trafficking, endocytosis

## Abstract

The cell interaction, mechanism of cell entry and intracellular fate of surface decorated nanoparticles are known to be affected by the surface density of targeting agents. However, the correlation between nanoparticles multivalency and kinetics of the cell uptake process and disposition of intracellular compartments is complicated and dependent on a number of physicochemical and biological parameters, including the ligand, nanoparticle composition and colloidal properties, features of targeted cells, etc. Here, we have carried out an in-depth investigation on the impact of increasing folic acid density on the kinetic uptake process and endocytic route of folate (FA)-targeted fluorescently labelled gold nanoparticles (AuNPs). A set of AuNPs (15 nm mean size) produced by the Turkevich method was decorated with 0–100 FA-PEG_3.5kDa_-SH molecules/particle, and the surface was saturated with about 500 rhodamine-PEG_2kDa_-SH fluorescent probes. In vitro studies carried out using folate receptor overexpressing KB cells (KB^FR-high^) showed that the cell internalization progressively increased with the ligand surface density, reaching a plateau at 50:1 FA-PEG_3.5kDa_-SH/particle ratio. Pulse-chase experiments showed that higher FA density (50 FA-PEG_3.5kDa_-SH molecules/particle) induces more efficient particle internalization and trafficking to lysosomes, reaching the maximum concentration in lysosomes at 2 h, than the lower FA density of 10 FA-PEG_3.5kDa_-SH molecules/particle. Pharmacological inhibition of endocytic pathways and TEM analysis showed that particles with high folate density are internalized predominantly by a clathrin-independent process.

## 1. Introduction

Recently, significant efforts have been dedicated towards developing active targeted nanocarriers for site-selective delivery of therapeutics. Though many successful preclinical trials have been achieved, none of the active targeted nanosystems have been approved for clinical use [1,2]. Several preclinical studies have shown that specific cell targeting is crucial to enhance the therapeutic profile of several drugs and yield intracellular bio-transport in the case of macromolecules, such as nucleic acids, toxins and theranostics that possess low permeability across the plasma membrane barrier [1,3]. Thus, peculiar receptors overexpressed on the plasma membrane of specific tissues, such as tumours, can be exploited for cell recognition and intracellular delivery of targeted nanocarriers via endocytic processes. Antibody-drug conjugates (ADCs) represent a typical example of active targeting since this can be achieved by antibody recognition of antigens, receptors or transporters on cell surfaces. However, cell uptake is not always achieved as noted for trastuzumab, an FDA-approved humanized monoclonal antibody against Her-2/neu tyrosine kinase receptor, that induces quite limited HER-2 receptor internalization in Her-2/neu positive metastatic breast cancer models [4,5,6,7,8,9], yielding good cell targeting but poor cell uptake. To enhance the cell uptake of targeted nanocarriers, accelerated internalization has been observed upon cross-linking the cell membrane bound antibody–receptor complex using an avidin/streptavidin–biotin system [10] and subsequent clustering of the receptor on plasma membrane to drive internalization. Also clustering of GPI-anchored proteins, such as folate receptor, results in their sequestration in caveolae and promotes caveolae-mediated endocytosis [11].

Studies performed with RGD peptide [12], HER-2 affibody [13], TAT peptide [14], oligonucleotide [15] and folate [16,17] targeted nanosystems have shown that cell interaction, avidity for the cell membrane receptor and uptake process are affected by the number and density of ligands on the particle surface [18]. Additionally, the multivalency property of the nanocarriers, namely, the density of the targeting agent on their surface, results in different cell uptake mechanisms and trafficking to subcellular compartments, which is also affected by nanoparticle size, shape and surface composition [19]. This can have significant implications on the efficacy of the therapeutic system. Thus, dedicated design of nanocarriers must be undertaken to rationally plan the most suitable intracellular trafficking pathway(s) and the resulting subcellular disposition of the drug nanocarriers especially for systems engineered for controlled release of the therapeutics within the intracellular environment. 

It is known that the uptake mechanisms of multivalent nanocarriers often differ from that of the respective monovalent ligand. As an example, receptor-mediated endocytosis of folic acid occurs via clathrin-independent endocytosis [20,21,22], while folate decorated nanocarrier uptake occurs via clathrin- or both clathrin- and caveolae-mediated endocytosis [23,24,25,26,27,28]. The data reported in the literature indicate that while the density of folate on nanocarrier surface dictates the uptake pathway [29], very little is known about the specific endocytic mechanisms involving multivalent nanocarriers. In particular, there is a paucity of information on the comparative kinetics of the uptake of folate and multivalent nanocarriers with increasing folate density. Indeed, the kinetics of the intracellular trafficking to specific compartments, typically endosomes and lysosomes, is crucial to allow design of nanocarriers with suitable “spatially-and-timely” controlled activation of drug release by, for example, chemical or enzymatic processes in subcellular compartments. 

Here, the intracellular trafficking kinetics to lysosomes and the process time for a range of folate decorated gold nanoparticles with variable multivalency has been comparatively investigated. 

Our studies were performed by using gold nanoparticles (AuNPs) that, due to thiol-based conjugation chemistry, allow for well-defined coating process with folate and labelling end-tipped PEGs with fluorophores. The study anticipates the exploitation of AuNPs as carriers of surface-associated drugs for intracellular controlled release.

## 2. Materials and Methods

### 2.1. Materials 

Folic acid (code F7876), sodium citrate tribasic dihydrate (code S4641), tetrachloroauric(III) acid trihydrate (code 520918), iodine (code 326143), potassium iodine (code 221945), barium chloride (code 342920), dimethyl sulfoxide, tris(2-carboxyethyl)phosphine hydrochloride (TCEP) (code 580560), triethylamine, dimethylsulfoxide anhydrous (DMSO), chloroform, dichloromethane (DCM), diethyl ether (Et_2_O), were purchased from Sigma-Aldrich (St. Louis, MO, USA). Cysteine, N-hydroxysuccinimide (NHS), N,N′-Dicyclohexylcarbodiimide and 5,5′-di-thiobis-(2-nitrobenzoic acid) (DTNB) were purchased from Fluka (Buchs, Switzerland). Analytical thin-layer chromatography (TLC) was carried out on glass sheets coated with silica gel (Merck F-254, Merck, Darmstadt, Germany). Sephadex G-25 superfine and Sephadex LH 20 gel filtration resins were obtained from Amersham Pharmacia Biotech (Uppsala, Sweden). In total, 2 kDa and 3.5 kDa Amino-mercapto PEG (NH_2_-PEG_2kDa_-SH and NH_2_-PEG_3.5kDa_-SH; purchase code A5143-1/HS-PEG2000-NH2HCl, A5144-1/HS-PEG3500-NH2HCl, respectively) were purchased from JenKem Technology USA (Plano, TX, USA). In total, 2 kDa methoxy-mercapto PEG (mPEG_2kDa_-SH, code PEG1169) was acquired from Iris Biotech GmbH (Marktredwitz, Germany). Carboxytetramethylrhodamine N-succinimidyl ester (Rhodamine-NHS, code C2211) was purchased from Thermo Fisher Scientific (Waltham, MA, USA). All the chemical reagents for cell culture, Dulbecco’s modified Eagle’s medium (DMEM) and folic acid free DMEM, RPMI-1640 medium, L-Glutamine solution (code G7513), D-(+)-Glucose solution (code G8644), sodium bicarbonate solution, foetal bovine serum (FBS, code F7524), penicillin-streptomycin solution, Trypan Blue solution, Dynasore (code 324410), fibronectin (code 10838039001) and trypsin (code T4174) were supplied by Sigma-Aldrich (St. Louis, MO, USA). In total, 10 kDa Dextran labelled with Alexa Fluor 647 (Dex647, code D22914) and Transferrin labelled with Alexa Fluor 488 (Tf488, code T13342) were purchased from Thermofisher (Waltham, MA, USA).

KB^FR-high^ (human epithelial cervix carcinoma cell line) and MCF7^FR-low^ (human breast adenocarcinoma cell line) cells were obtained from the American Type Culture Collection.

### 2.2. Synthesis of Folate-PEG_3.5kDa_-SH (FA-PEG_3.5kDa_-SH) 

FA-PEG_3.5kDa_-SH was synthesized as reported by our group with slight modifications [30]. Briefly, folic acid (50.0 mg, 0.113 mmol) was dissolved in 1 mL of anhydrous DMSO. N-hydroxysuccinimide (NHS—15.6 mg, 0.136 mmol) was added to the solution, followed by dicyclohexylcarbodiimide (DCC—28.1 mg, 0.136 mmol). The mixture was stirred for 18 h at room temperature in the dark and then filtered using a sintered glass funnel. The N-Hydroxysuccinimidyl-ester-activated folic acid was isolated by precipitation in diethyl ether and dried under reduced pressure and used without purification for the following step. N-hydroxysuccinimidyl-ester-activated folic acid (25 mg, 0.046 mmol) and NH_2_-PEG_3.5kDa_-SH (54.1 mg, 0.015 mmol) were dissolved in 1 mL of anhydrous DMSO containing triethylamine (1.51 mg, 0.015 mmol). The reaction mixture was stirred for 12 h at room temperature in the dark, and then the crude product was isolated by precipitation in diethyl ether (40 mL) and purified by size exclusion chromatography using a Sephadex G-25 superfine resin eluted with ammonia solution (pH 9) as mobile phase. The eluted fractions were tested by UV-Vis spectroscopy at 363 nm and the Iodine test [31] for folate and PEG detection, respectively, and the positive ones of both tests (Absorbance above 0.1) were pooled together and freeze-dried. 

The product containing FA-PEG_3.5kDa_-S-S-PEG_3.5kDa_-FA and FA-PEG_3.5kDa_-SH (20 mg, 8.2 µmoles) was dissolved in 50 mM acetate pH 5, followed by Tris(2-carboxyethyl)phosphine (TCEP—20.5 mg, 82 µmoles), and stirred for 3 h. The mixture was dialysed against 1 mM HCl, 1 mM EDTA for 2 days using a 1 kDa MWCO Spectra/Por Float-a-lyzer G2 and freeze-dried. The final product FA-PEG_3.5kDa_-SH was dissolved in phosphate buffer at pH 7.4 and characterized by UV-Vis spectrophotometric analysis (l_max_ = 363 nm, ε_363_ = 6.197 M^−1^ cm^−1^) and Iodine assay [31]. The final recovery yield was 71%. The conjugation efficiency was calculated according to the following Equation (1):

(1) $$\;\mathrm{conjugation}\;\mathrm{efficiency}\;\left(\%\right)=\frac{\left(Abs_{363\;\mathrm{nm}}/6197{\;M}^{-1}{\mathrm{cm}}^{-1}\right)}{\mathrm{PEG}\;\left(M\right)}\times 100$$

FA-PEG_3.5kDa_-SH was further characterized by DTNB assay [32], MALDI mass spectrometry and RP-HPLC using a chromatographic system equipped with a Phenomenex RP-C18 Luna column (Torrance, CA, USA) eluted with 10 mM ammonium acetate, pH 6.5 (eluent A) and acetonitrile (eluent B), in a gradient mode from 10 to 40% of eluent B in 40 min, using a UV-Vis detector set at 363 nm. 

### 2.3. Synthesis of Rhodamine-PEG_2kDa_-SH (Rho-PEG_2kDa_-SH)

Rhodamine-NHS (25 mg, 47.3 µmol) was dissolved in anhydrous DMSO (500 µL) under stirring and containing NH_2_-PEG_2kDa_-SH (78.9 mg, 39.4 µmol) in the presence of triethylamine (3.99 mg, 39.4 µmol). The reaction was carried out for 18 h under stirring at room temperature in the dark. Afterwards, the mixture was purified by size-exclusion chromatography using Sephadex LH 20 resin and ethanol as eluent. The column fractions were collected and analysed by UV-Vis spectrophotometric measurement at 552 nm and Iodine test (535 nm) to detect Rhodamine and PEG, respectively. Fractions positive to both tests were pooled and the solvent removed under reduced pressure. The product was re-dissolved in 1 mM HCl and freeze-dried to obtain a pink powder. A 1 mg/mL solution of rhodamine-PEG_2kDa_-SH was prepared in 10 mM bicarbonate pH 9 and analysed by UV-Vis spectrophotometry (l_max_ = 552 nm, e_M_ = 80,000 cm^−1^M^−1^) and Iodine test to quantify Rhodamine and PEG concentration, respectively. The conjugation yield was calculated according to Equation (2):

(2) $$\mathrm{conjugation}\;\mathrm{efficiency}\;\left(\%\right)=\frac{\left(Abs_{552\;\mathrm{nm}}/80,000{\;M}^{-1}{\mathrm{cm}}^{-1}\right)}{\mathrm{PEG}\;\left(M\right)}\times 100$$

To reduce possible disulphide molecules (rhodamine-PEG_2kDa_-S-S-PEG_2kDa_-rhodamine), the product (20 mg, 8.28 µmol) was dissolved in 1 mL of 50 mM acetate pH 5 and Tris(2-carboxyethyl)phosphine (TCEP, 20.73 mg, 82.85 µmoles) was added to the solution. The reaction was carried out for 3 h under stirring at room temperature. The rhodamine-PEG_2kDa_-SH was then dialysed for 24 h against 1 mM HCl, 1 mM EDTA using a 1 kDa MWCO Spectra/Por Float-a-lyzer G2. The final recovery yield was 65%. The product was lyophilized and characterized by MALDI-TOF mass spectrometry and Ellman assay to assess the percentage of free thiol groups.

### 2.4. Synthesis of Folate-Pentane-Rhodamine (FA-C_5_-Rho)

The conjugate FA-C_5_-Rho was synthesized according to a three-step procedure. Folic acid was first activated as N-Hydroxysuccinimidyl-ester, as described in the “Synthesis of folate-PEG_3.5kDa_-SH” section. 

1,5-Diaminopentane (C_5_—130.5 mg, 1.28 mmol) was dispersed in 150 µL of anhydrous DMSO. N-Hydroxysuccinimidyl-ester-activated Folic acid (FA-NHS—23.0 mg, 0.04 mmol) was dissolved in anhydrous DMSO (1 mL) in the presence of triethylamine (TEA—178.1 µL, 1.28 mmol) and added dropwise to the 1,5-Diaminopentane solution. The reaction was left to proceed for 18 h under stirring at room temperature in the dark. The crude product was isolated by precipitation in 40 mL of Et_2_O to remove unreacted 1,5-Diaminopentane. The crude product containing folate-5-aminopentane (FA-C_5_-NH_2_) conjugate was washed with Et_2_O (3 × 40 mL) and then dried under vacuum. FA-C_5_-NH_2_ (16.6 mg, 31.56 µmol) was dissolved in anhydrous DMSO (1 mL) containing TEA (3.83 mg, 37.88 µmol). Rhodamine-NHS (700 µL of a 54.1 mM solution) in anhydrous DMSO was added dropwise to the FA-C_5_-NH_2_ solution and left for 18 h whilst stirring at room temperature in the dark. The crude product was recovered by precipitation in diethyl-ether, the solid was redissolved in 500 µL of DMSO and purified by size exclusion chromatography using a Sephadex LH20 resin eluted with ethanol. The collected fractions were analysed for FA and Rhodamine content by UV-Vis absorbance at 363 nm and 552 nm, respectively, and the positive ones to both wavelengths (Absorbance above 0.1) were pooled together, and the organic solvent was removed by reduced pressure. The solid was re-dissolved in milliQ water and lyophilized, yielding FA-C_5_-Rho as a pink powder (18.2 mg, 19.4 µmol, 61% of yield) that was characterized by ESI-TOF mass spectrometry.

FA-C_5_-NH_2_ theoretical MW for [M-H]^−^ = 526.2531 Da, experimental m/z for [M-H]^−^ = 526.2525.

FA-C_5_-Rho theoretical MW for [M]^+^ 940.4106 Da, experimental m/z for [M]^+^ = 940.4101.

### 2.5. Gold Nanoparticle (AuNP) Preparation 

AuNPs were prepared according to the Turkevich method by reduction of tetrachloroauric acid with sodium citrate [33]. Size and particle concentration were assessed by Dynamic Light Scattering and UV-Vis spectrophotometry, as reported by our group [30,34].

### 2.6. AuNP Surface Decoration Efficiency 

AuNPs were incubated with increasing amounts of each different thiolated PEG derivative to assess the conjugation efficiency. FA-PEG_3.5kDa_-SH, Rhodamine-PEG_2kDa_-SH, mPEG_2kDa_-SH aqueous solutions (5 mg/mL) were prepared, and different volumes of each polymer solution were added separately to 10 mL of 3 nM AuNP suspensions to achieve FA-PEG_3.5kDa_-SH/AuNPs, rhodamine-PEG_2kDa_-SH/AuNPs and mPEG_2kDa_-SH/AuNPs molar ratios in the 10:1–100:1, 10:1–2000:1 and 10:1–2000:1 ranges, respectively. Control samples with identical polymer concentration in the absence of AuNPs were prepared.

Formulations were placed in a rotating shaker and left under stirring for 8 h at room temperature. Modified AuNPs were then pelleted by centrifugation at 14,500× *g* for 30 min. To detect unbound PEG derivatives: (1) supernatants of samples containing FA-PEG_3.5kDa_-SH were tested by Iodine assay and spectrophotometric UV-Vis analysis at 363 nm; (2) supernatants of samples decorated with rhodamine-PEG_2kDa_-SH were tested by Iodine assay and spectrophotometric analysis at 552 nm; (3) supernatants of AuNPs modified with mPEG_2kDa_-SH were tested by Iodine assay only. Decoration efficiency % of each formulation was derived using the following Equation (3):

(3) $$\mathrm{decoration}\;\mathrm{efficiency}\;\left(\%\right)=\frac{\left(\mathrm{fed}\;\mathrm{PEG}\;-\;\mathrm{unbound}\;\mathrm{PEG}\right)}{\mathrm{fed}\;\mathrm{PEG}\;}\times 100$$

### 2.7. Preparation of Fluorescent Folate Decorated AuNPs (Rho-FA_x_-AuNPs)

A library of FA-targeted rhodamine-labelled AuNPs were produced at increasing FA-PEG_3.5kDa_-SH density, ranging from 10:1 to 100:1 FA-PEG_3.5kDa_-SH/AuNP feed molar ratio, and at a fixed rhodamine-PEG_2kDa_-SH/AuNP feed molar ratio of 1000:1. 

As an example, the preparation of fluorescent 100:110:1000:1 FA-PEG_3.5kDa_-SH/mPEG_2kDa_-SH/rhodamine-PEG_2kDa_-SH/AuNP formulations is described below.

FA–PEG_3.5kDa_-SH aqueous solution (23.6 µL of 0.5 mg/mL) was mixed with 12 µL of 50 µg/mL mPEG_2kDa_-SH aqueous solution. The polymer mixture was added to a 3 nM gold nanoparticle suspension (10 mL) to provide a 100:10:1 FA–PEG_3.5kDa_-SH/mPEG_2kDa_-SH/AuNP molar ratio, and the suspension was placed in a rotary mixer and left under stirring for 18 h at room temperature. Then, an aliquot (1 mL) of the mixture was centrifuged at 14,500× *g* for 30 min at 4 °C to isolate the particles, and the supernatant underwent spectrophotometric analysis at 363 nm to detect the unbound FA-PEG_3.5kDa-_SH. Subsequently, 14.5 µL of a 5 mg/mL Rho-PEG_2kDa_-SH solution in milliQ water (2.07 µmol) and 12 µL of 0.5 mg/mL mPEG_2kDa_-SH aqueous solution were added to the particle suspension, yielding a 1000:100:1 Rho–PEG_2kDa_-SH/mPEG_2kDa_-SH/AuNP molar ratio. The resulting decorated particles were recovered by centrifugation at 14,500× *g* for 30 min at 4 °C. AuNPs were resuspended in 9 mL of milliQ water and analysed by Dynamic Light Scattering (DLS) and by UV-Vis spectrophotometry in the 200–600 nm range to assess the particle concentration, as described by Liu and co-workers [35]. The supernatant underwent UV-Vis absorbance analysis at 552 nm to assess unbound Rho-PEG_2kDa_-SH. 

The other members of the AuNP library at lower FA-PEG_3.5kDa_-SH density were generated with the same procedure by replacing the FA-PEG_3.5kDa_-SH with mPEG_2kDa_-SH. 

Control non-targeted particles (Rho-FA_0_-AuNPs) were produced using the same procedure by replacing Folate-PEG_3.5kDa_-SH with mPEG_3.5kDa_-SH.

### 2.8. Decorated AuNP Size and Morphology

Size analysis. The size of decorated AuNPs was measured at 25 °C with a Dynamic Light Scattering Zetasizer Nano (Malvern Panalytical Ltd., Malvern, UK). DTS version 6.12 software (DTS Software, Raleigh, NC, USA) was used to analyse the data. Sizes were referred to by intensity and number average. Each analysis resulted from three measurements with a fixed 10 runs per 10 s. 

### 2.9. Transmission Electron Microscopy (TEM) Imaging

TEM images were obtained with a Tecnai G2 microscope (FEI, Hillsboro, OR, USA). Freshly prepared non-coated particles in milliQ water (2 nM) were placed on a carbon-coated copper grid, and the water was allowed to dry at room temperature. Functionalized AuNPs were negatively stained with 1% *w*/*v* uranyl acetate in distilled water before imaging.

The average diameter of particles was calculated by measuring 200 individual particles with ImageJ software version 1.51j8 (National Institutes of Health software package by Wayne Rasband; Bethesda, MD, USA). 

### 2.10. Cell Culture

KB^FR-high^ cells (human cervical carcinoma) [36] were grown at 37 °C, in 5% CO_2_ humidified atmosphere, using folic-acid-free DMEM medium supplemented with 15% heat inactivated FBS, 2 mM L-glutamine, 100 IU/mL penicillin, 100 μg/mL streptomycin and 0.25 μg/mL of amphotericin B (Sigma-Aldrich, St. Louis, MO, USA). MCF-7^FR-low^ (human breast adenocarcinoma) [36] were grown at 37 °C, in 5% CO_2_ atmosphere, using RPMI-1640 medium supplemented with 10% FBS, 100 IU/mL penicillin, 100 μg/mL streptomycin and 0.25 μg/mL of amphotericin B. Cells were routinely harvested by incubation with 0.125 mg/mL trypsin for 5 min at 37 °C and seeded in new 75 cm^2^ flasks.

### 2.11. AuNP Association with KB^FR-high^ and MCF-7^FR-low^ Cells 

Quantification of AuNP association with cells was monitored by Atomic Absorption Spectroscopy and Flow Cytometry.

#### 2.11.1. Atomic Absorption Spectroscopy

MCF-7^FR-low^ and KB^FR-high^ cells in Folic-free DMEM medium (FFDMEM) containing 15% foetal bovine serum (FBS) were seeded in 12-well plates at a density of 5 × 10^5^ cells per well and grown for 24 h. Then, the medium was removed, cells were washed with 1 mL PBS and incubated with 1 mL of FFDMEM containing 2 nM rhodamine labelled folate-targeted AuNPs (Rho-FA_x_-AuNPs), and cells were incubated for 2 h at 37 °C. Afterwards, the particle-containing medium was removed, and the cells were washed thrice with PBS without Mg^2+^/Ca^2+^. Cells were then detached by 0.125 mg/mL trypsin treatment (150 µL per well). The trypsin activity was quenched by adding 500 µL of PBS with Mg^2+^/Ca^2+^, and samples were centrifuged at 73× *g* for 5 min. Cell pellets were washed twice with PBS and then added to 600 µL of 0.1% *v*/*v* Triton^®^ X-100 in water and incubated for 15 min under gentle shaking at room temperature. Cell lysates (500 µL) were digested by aqua regia treatment (1:3 HNO_3_/HCl *v*/*v*, 5 mL) at 80 °C for 1 h. Gold quantification was performed by Atomic Absorption Spectrometry (AAS) using a Varian AA240 Zeeman instrument equipped with a GTA120 graphite furnace, Zeeman background corrector and an autosampler (Varian Inc., Palo Alto, CA, USA). The gold content was normalized by the number of cells, which was derived by BCA Protein Assay (Thermo Fisher Scientific Inc., Waltham, MA, USA) performed on 100 µL of the cell lysate.

#### 2.11.2. Flow Cytometric Analysis

KB^FR-high^ cells were seeded in 12-well plates at the density of 3 × 10^5^ cells per well and allowed to adhere for 24 h. The medium was removed, cells were washed with 1 mL of PBS and incubated with 1 mL of 2 nM Rho-FA_x_-AuNPs in FFDMEM, and cells were incubated for 2 h at 37 °C in a humidified 5% CO_2_ atmosphere. Afterwards, cells were washed with PBS without Ca^2+^/Mg^2+^ (3 × 1 mL/well) and detached by treatment with 0.125 mg/mL trypsin in PBS without Ca^2+^/Mg^2+^ (150 μL/well). After 5 min of incubation at 37 °C, trypsin activity was quenched by addition of 500 μL/well of PBS with Mg^2+^/Ca^2+^, and cells were recovered by centrifugation at 73× *g* for 5 min. Supernatants were removed, and the cell pellet was fixed by incubation with freshly prepared 4% *w*/*v* paraformaldehyde (PFA) in PBS for 15 min at 4 °C. Samples were centrifuged at 73× *g* for 5 min, washed with PBS and analysed by flow cytometry. Cell samples were analysed at λ_ex_ 550 and λ_em_ 575 for Rhodamine detection. 

In both experiments, competition assay was performed as described above by incubating KB^FR-high^ cells with Rho-FAx-AuNP suspensions in FFDMEM containing 200 μM folic acid.

#### 2.11.3. Confocal Microscopy

KB^FR-high^ cells were seeded onto 35 mm imaging dishes (MatTek, Ashland, MA, USA) at a density of 1.5 × 10^5^ cell per well in FFDMEM added to 15% *v*/*v* FBS and grown for 48 h at 37 °C and 5% CO_2_. After washing cells with 0.5 mL PBS, Rho-FA-AuNPs (250 μL, 2 nM) in FFDMEM at pH 7.4 were added to each dish, and cells were incubated at 37 °C in the dark for 2 h. Competition assay was performed by incubating KB^FR-high^ cells with 0.5 mL of Rho-FA_x_-AuNP suspensions in FFDMEM containing 200 μM folic acid. 

FA-C_5_-Rho was also tested with KB^FR-high^ cells as monovalent control. Cells were incubated with 100 nM FA-C_5_-Rho, corresponding to the equimolar concentration of folate in Rho-FA_50_-AuNPs, with 50 units of Folate-PEG_3.5kDa_-SH per AuNPs. The particle-containing medium was then removed, and wells were gently washed three times with PBS. Cells were incubated in pre-warmed imaging medium (phenol red-free DMEM, pH 7.4 containing 25 mM HEPES and supplemented with 1 mg/mL BSA) and imaged live on a Leica SP5 confocal laser-scanning microscope equipped with a 488 nm Ar laser, 543/633 nm HeNe laser, 100 × 1.4 NA or 40 × 1.25 NA objectives using a Leica Type F immersion oil. Cell samples were irradiated with the 514 nm laser for Rhodamine detection. Captured confocal images were analysed by ImageJ script 1.47v (National Institutes of Health software package by Wayne Rasband; Bethesda, MD, USA). The selection of fluorescent pixels was performed after setting an ImageJ IsoData threshold to derive the fluorescence intensity. The background intensity was set by looking at pixels that showed a fluorescence intensity below the IsoData threshold. The fluorescence intensity correction of each image was performed by subtracting the background intensity from the intensity value. Corrected mean intensities were calculated as the mean intensity value of 5 images for each sample. All the fluorescence intensity values were normalized to the relative maximum corrected mean intensity value. The mean normalized intensity from 3 independent experiments was plotted.

#### 2.11.4. Transmission Electron Microscopy (TEM) of Cells

KB cells were seeded at a density of 3 × 10^5^ cells per well in 12-well plates. After 24 h, cells were incubated with 2 nM Rho-FA_x_-AuNPs in FFDMEM for 2 h at 37 °C. The medium was then removed, and the cells washed three times with PBS prior to fixing with 2.5% *w*/*v* glutaraldehyde in 0.1 M sodium cacodylate at 4 °C for 1 h. Cells were washed twice with sodium cacodylate buffer and post fixed in 1% *w*/*v* osmium tetroxide in 0.1 M sodium cacodylate for 1 h. Dehydration of cell samples was performed in a graded series of ethanol solutions, followed by embedding in fresh EPON resin. Ultrathin 70 nm sections of the samples were cut and observed with Tecnai G2 Transmission Electron Microscope (FEI, Hillsboro, OR, USA).

### 2.12. Intracellular Trafficking Studies

#### 2.12.1. Kinetic Analysis of AuNP Trafficking to Lysosomes

KB cells were seeded on 35 mm imaging dishes at a density of 1.5 × 10^5^ cell per dish in FFDMEM containing 15% *v*/*v* Foetal Bovine Serum (FBS) and grown for 48 h at 37 °C and 5% CO_2_. Cells were ‘pulsed’ for 8 h with Dex647 (50 µg/mL in FFDMEM supplemented with 15% *v*/*v* FBS) to allow traffic to lysosomes then ‘chased’ for 12 h in dextran-free FFDMEM complete medium to allow traffic to and enrichment in lysosomes, according to the procedure reported by Jones and co-workers [37]. To study particle uptake into lysosome labelled cells, cells were then incubated for 30 min with 2 nM of Rho-FA_10_-AuNPs or Rho-FA_50_-AuNPs, or 100 nM FA-C_5_-Rho solution in FFDMEM medium. Afterwards, the medium was removed, cells were washed thrice with PBS, incubated in pre-warmed imaging medium at 37 °C, 5% CO_2_ atmosphere and imaged at scheduled times (0, 1, 2 and 4 h post-treatment) via confocal microscopy using the 543 later and 633 lasers for Rhodamine and Alexa647 analysis, respectively.

Analysis of each field was performed using an ImageJ script without manual intervention. A Li threshold was applied to the Dex647 channel to identify the lysosomal regions. To calculate the intensity of rhodamine in the lysosomes was determined according to Moody et al. [8]. For this, a mask of the lysosomal region was initially created using a Li threshold of the Dex647 channel. The mean intensity of rhodamine in this region was calculated using this mask, and the mean background intensity in the Rhodamine channel (calculated as above using the IsoData threshold) was subtracted from this value. This lysosomal intensity value was calculated for 5 separate images and averaged to get a mean intensity value for each time point and condition within one experiment. These values were normalised against the highest value within one experiment, and the mean was taken from three independent experiments.

The colocalization kinetic of Rho-FA_10_-AuNPs and Rho-FA_50_-AuNPs, and the conjugate FA-C_5_-Rho with Dex647 labelled lysosomes was evaluated by Pearson’s coefficient (PC) at the scheduled timepoints (0, 1, 2 and 4 h post-treatment). Images per time point (5) were captured and analysed, and to account for intercellular variation, 5 individual cells were manually selected for each image. PC values were calculated using the JaCOP ImageJ plugin, and the mean PC value of 5 cells per 5 different images per time point was calculated. Standard deviation was calculated as variation between mean PC values of 3 independent experiments.

#### 2.12.2. Uptake Pathway Inhibition Assay

KB cells were seeded onto 35 mm imaging dishes at a density of 1.5 × 10^5^ cell per dish in FFDMEM supplemented with 15% *v*/*v* FBS and grown for 48 h at 37 °C and 5% CO_2_. Cells were then preincubated for 30 min at 37 °C with 80 µM Dynasore in FFDMEM without serum. The medium was then removed, and cells were incubated for 30 min with 2 nM Rho-FA_50_-AuNPs in FFDMEM without FBS or with 10 µg/mL AlexaFluor 488 labelled Transferrin (Tf-488) in FFDMEM without FBS, in the presence or absence of Dynasore (80 µM). After incubation, the medium was removed, cells were washed 3 times with PBS and fixed with 4% *w*/*v* PFA in PBS for 15 min. Cells were washed twice with PBS and then analysed by confocal microscopy using a 514 nm laser for Tf488 and Rho-FA_50_-AuNP detection. 

### 2.13. Statistical Analysis 

All experiments were carried out three times, and each sample was generated in triplicate. Data are presented as mean ± S.E. calculated from three independent experiments. Statistical analyses were performed with GraphPad software. Statistical comparisons between treatment groups were performed with analysis of variance (two-way ANOVA), and the threshold of significance was calculated according to Bonferroni’s test. Statistical significance was attained for values of *p* < 0.05.

## 3. Results and Discussion

Gold nanoparticles (AuNPs) were surface decorated with different amounts of folic acid to investigate the effect of targeting agent density on nanoparticle uptake efficiency and intracellular trafficking profile on folate receptor overexpressing tumour cells. Folic acid was anchored on the AuNP surface by using Folate-PEG_3.5kDa_-SH, while fluorescent AuNPs were obtained by using Rhodamine-PEG_2kDa_-SH. Rhodamine was conjugated to the AuNP surface by using a shorter PEG spacer with respect to Folate-PEG_3.5kDa_-SH in order to avoid the folic acid masking. 

### 3.1. Synthesis and Characterization of Thiolated Derivatives for AuNP Decoration and Fluorescent Folate

#### 3.1.1. Folate-PEG3.5kDa-SH Synthesis

**Scheme 1:**
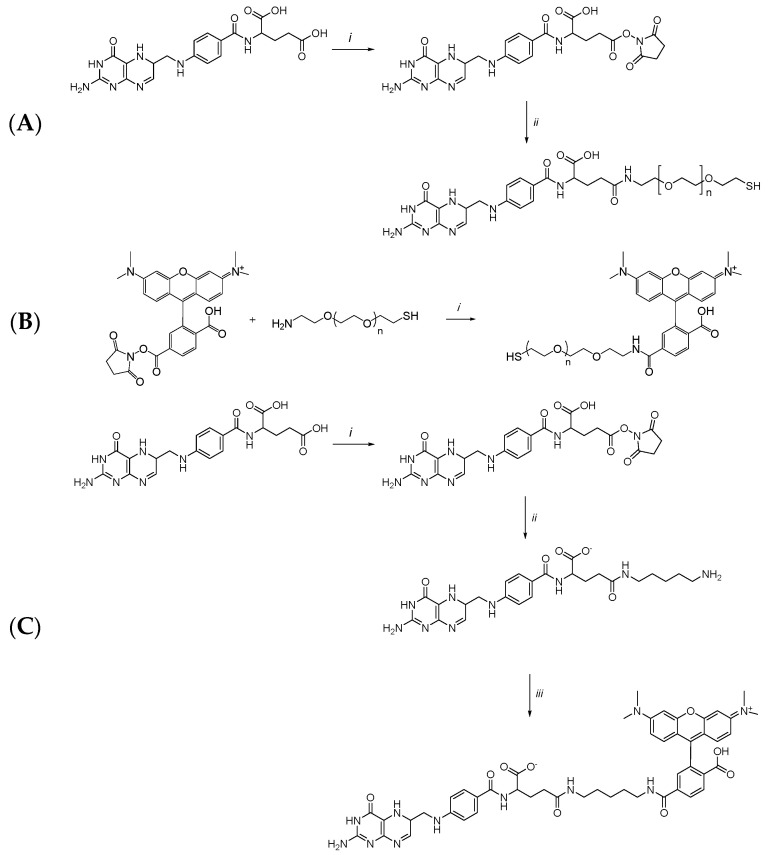
(**A**) Synthesis of FA-PEG_3.5kDa_-SH—I. Reagents and conditions: (i) NHS, DCC, anhydrous DMSO, 18 h, room temperature, in the dark; (ii) NH_2_-PEG_3.5kDa_-SH, TEA, anhydrous DMSO, 12 h, room temperature, in the dark. (**B**) Synthesis of Rhodamine-PEG_2kDa_-SH. Reagents and conditions: (i) anhydrous DMSO, TEA, 18 h, room temperature, in the dark. (**C**) Synthesis of FA-C_5_-Rho. Reagents and conditions: (i) NHS, DCC, anhydrous DMSO, 18 h, room temperature, in the dark; (ii) 1,5-Diaminopentane, anhydrous DMSO, TEA, 18 h, room temperature, in the dark; iii. Rhodamine-NHS, anhydrous DMSO, TEA, 18 h, room temperature, in the dark.

To yield adequate exposure of the targeting ligand (folic acid, FA) on the gold nanoparticle (AuNP) surface, FA was conjugated through its γ-COOH group to a flexible and hydrophilic thiol terminating NH_2_-PEG_3.5kDa_-SH, according to an established procedure [38] and as reported in Scheme 1A. The adopted protocols yielded efficient conjugation of FA to NH_2_-PEG_3.5kDa_-SH, which was found to be 98 mol/mol%.

Importantly, folic acid displays two carboxylic groups in the g- and α-position of the glutamate moiety, and the α-carboxyl site is involved in the folate receptor recognition (Kd~10^−9^ M) [39]. Acid activation was performed by using a low molar excess of coupling agents with respect to FA (DCC/NHS/FA 1.2:1.2:1) to favour the conjugation of FA to the polymer through the less hindered and most accessible g-carboxylic group [40]. The Ellman’s assay performed after the conjugate reduction by TCEP showed that 97 mol/mol% of thiol groups of FA-PEG_3.5kDa_-SH were available. MALDI-TOF mass spectrometry (Figure S1) and reverse phase high-performance liquid chromatography analyses of FA-PEG_3.5kDa_-SH confirmed the chemical identity and mol wt (4100 Da) of the conjugate and the absence of free FA.

#### 3.1.2. Rho-PEG2kDa-SH Synthesis

Fluorescent FA-targeted AuNPs were prepared to track the association of the conjugates with and in cells. Rhodamine-NHS was conjugated via standard coupling reaction to NH_2_-PEG_2kDa_-SH (Scheme 1B) with 96 mol% conjugation yield, as confirmed by MALDI-TOF analysis of the Rho-PEG_2kDa_-SH conjugate (Figure S2), showing a molecular weight around 2600 Da.

#### 3.1.3. Folate-1,5-diaminopentane-rhodamine (FA-C_5_-Rho) Synthesis

Folate-pentane-Rhodamine (FA-C_5_-Rho) was synthesized as reference molecule to investigate the biological behaviour of AuNP non-conjugated FA. The fluorescent FA derivative was synthesized by a three-step procedure illustrated in Scheme 1C: 1. FA activation to the corresponding N-hydroxysuccinimidyl-ester; 2. mono-conjugation of 1,5-diaminopentane to yield FA-NH_2_; 3. conjugation of Rho-NHS to FA-NH_2_.

A large excess of 1,5-diaminopentane was used in the second step to avoid the derivatization of both amino groups with folic acid (i.e., FA-C_5_-FA). The chemical identity FA-C_5_-NH_2_ and FA-C_5_-Rho was confirmed by ESI-TOF mass spectrometry showing the expected m/z for [M-H]^−^ of 526.2525 and m/z for [M]^+^ of 940.4101, respectively.

### 3.2. Gold Nanoparticle Production

**Figure 1:**
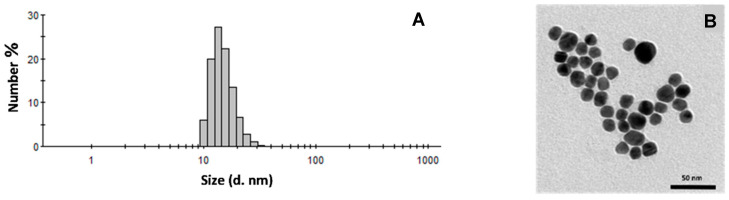

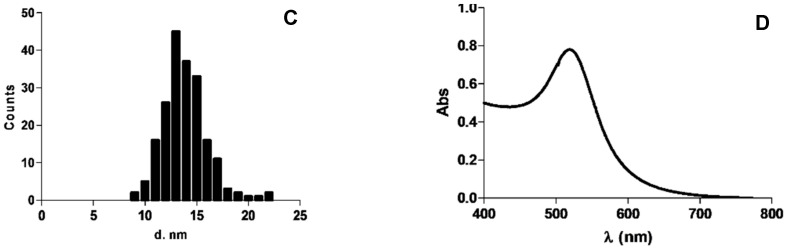
Size distribution profile of by Dynamic Light Scattering (**A**), TEM image (**B**), size distribution profile by ImageJ TEM image processing analysis (200 particles) (**C**), and visible spectrum (**D**) of freshly prepared citrate stabilized gold nanoparticles.

DLS analysis of gold nanoparticles (AuNPs) obtained by reduction of tetrachloroauric(III) acid (HAuCl_4_) according to the Turkevich’s method showed a size of 14.5 ± 1.6 nm in number, and 19.5 ± 3.8 in intensity and polydispersity index (PDI) of 0.20 ± 0.08 (Figure 1A), indicating the production protocol yielded homogenous size population. The TEM images analysis confirmed the DLS data (14.6 ± 2.3 nm mean size) and showed the spherical particles shape (Figure 1B,C).

The AuNP concentration derived by spectrophotometric analysis (Figure 1D) and calculated as described in the literature [35,41,42] resulted to be 3 nM. The size of particles from DLS analysis expressed as a number was used to calculate particle concentration, as requested by the Liu et al. equation [35].

The zeta potential analysis showed a slightly negative charge (−39.9 ± 3.3 mV), which was ascribed to the surface adsorbed citrate. Notably, since citrate interacts with metal gold with a strength comparable to that of the hydrogen bond (8−10 kcal/mol) [43], it can be easily displaced by ligands during the subsequent decoration step with thiol ligands, whose bond with metallic gold is almost 4 times stronger (40 kcal/mol) [44].

### 3.3. AuNP Surface Decoration

Several studies reported in literature highlight that multiple targeting ligands on nanoparticle surface can increase binding avidity, accelerate the internalization process and, thus, can be considered to enhance the therapeutic efficacy of a drug nanocarrier [45,46,47]. In order to investigate the effect of the targeting agent density on nanoparticle surface on targeting profile of the nanosystems, a library of Rho-FA_x-_AuNPs was produced at increasing density of FA-PEG_3.5kDa_-SH followed by gold surface saturation with Rho-PEG_2kDa_-SH.

Preliminarily studies were performed to investigate the AuNP decoration efficiency with fluorescent and targeting agent.

**Figure 2:**
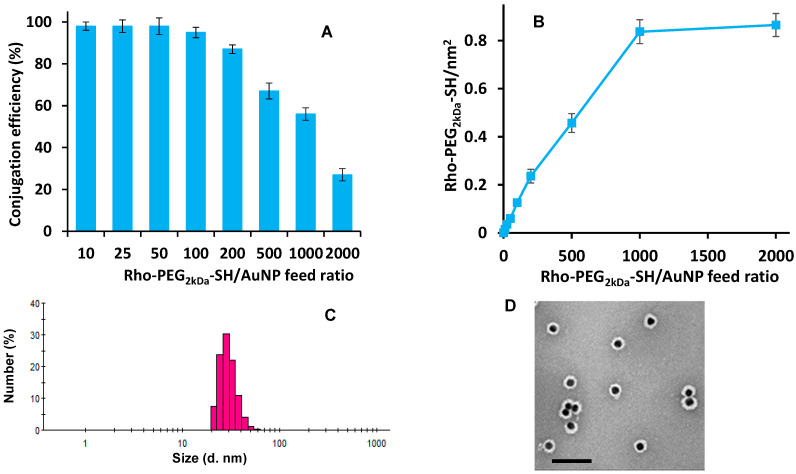
(**A**) Rho-PEG_2kDa_-SH conjugation efficiency profile to AuNPs. (**B**) Density of Rho-PEG_2kDa_-SH on AuNPs surface at increasing Rho-PEG_2kDa_-SH/AuNP feed molar ratio. (**C**) Size profile by DLS of Rho-FA_50_-AuNPs in milliQ water. (**D**) TEM image of Rho-FA_50_-AuNPs. Size bar = 100 nm.

The Rho-PEG_2kDa_-SH conjugation to AuNPs was investigated by using increasing Rho-PEG_2kDa_-SH/AuNP molar feed ratios (10:1–2000:1). The unbound fluorescent moiety was quantified in the supernatants by spectrofluorimetric analysis. The results reported in Figure 2A show a progressive decrease of the functionalization efficiency as the Rho-PEG_2kDa_-SH/AuNP molar feed ratio increased. Maximum saturation was achieved with 1000:1 Rho-PEG_2kDa_-SH/AuNP feed molar ratio (Figure 2B), corresponding to about 560 polymer chains per AuNPs. Considering the mean size and spherical shape of the AUNPs, the Rho-PEG_2kDa_-SH density on the AuNP surface was calculated to be about 0.8 chains/nm^2^.

According to previous studies showing that 50:1 FA-PEG_3.5kDa_-SH/AuNP feed molar ratio yielded AuNPs with excellent targeting behaviour [30,34], the FA-PEG_3.5kDa_-SH decorating conditions were tested in the range of 10:1–100:1 FA-PEG_3.5kDa_-SH/AuNP feed molar ratio. All the feed molar ratios tested resulted in nearly complete FA-PEG_3.5kDa_-SH binding (>95%) to the nanoparticle surface.

According to the preliminary studies, a series of AuNPs decorated with both targeting and fluorescent moieties (Rho-FA_x_-AuNPs), were prepared by using 0, 10, 25, 50, 100 FA-PEG_3.5kDa_-SH/AuNP feed molar ratio followed by surface saturation with 1000:1 Rho-PEG_2kDa_-SH/AuNP feed molar ratio.

Under these conditions, the saturation of AuNP surface resulted in 95% and about 50% of FA-PEG_3.5kDa_-SH and Rho-PEG_2kDa_-SH conjugation, respectively, corresponding to 0, 0.015, 0.037, 0.075 and 0.15 FA-PEG_3.5kDa_-SH chains/nm^2^ and about 0.7 Rho-PEG2_kDa_-SH chains/nm^2^ (Table S1).

The Rho-FA_x_-AuNPs showed similar size and PDI. The DLS analysis of the AuNPs decorated with 50 molecules of FA-PEG_3.5kDa_-SH (Rho-FA_50_-AuNPs) reported Figure 2C as representative of the library, showed a particle size of 29.3 ± 6.5 nm and a low PDI (0.31).

The TEM image reported in Figure 2D shows that the particle maintained spherical morphology, with an average size of 29.9 ± 3.2 nm (n = 200) and a homogeneous size distribution without the presence of aggregates, which is in agreement with the DLS analysis. Furthermore, the grey corona surrounding the particle core (black dots) confirms the presence of the polymeric chains and results from the low electron density of the organic material relative to the metallic core of the particles.

### 3.4. Cell Association and Uptake Studies

**Figure 3:**
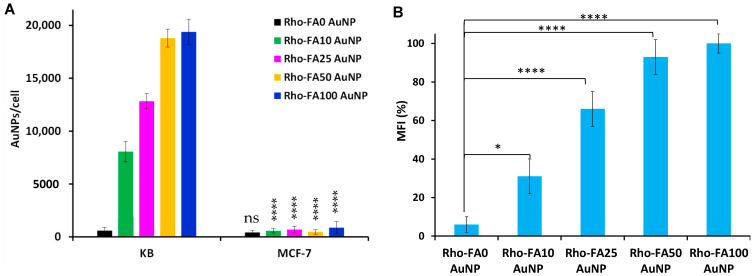
(**A**) Cell association profile of Rho-FA_x_-AuNPs by KB^FR-high^ and MCF-7^FR-low^ cells by atomic absorption analysis of cell lysates. Statistical significance was calculated versus corresponding uptake of MCF-7^FR-low^ cells: ns *p* > 0.05, **** *p* < 0.0001. (**B**) Mean Fluorescence intensity percentage of KB^FR-high^ cells incubated with Rho-FA_x_-AuNPs with increasing FA-PEG_3.5kDa_-SH density and non-targeted gold nanoparticles (Rho-FA_0_-AuNPs). The MFI were normalized to the MFI of cells incubated with Rho-FA_0_-AuNPs. Statistical significance was calculated versus non-targeted particles (Rho-Fa_0_-AuNPs): * *p* < 0.05, **** *p* < 0.0001.

The effect of ligand density on the Rho-FA_x_-AuNP association with target cells was investigated using KB^FR-high^ cells, a cancer cell line that overexpress the folate receptor (FR); MCF-7^FR-low^ cells with low expression of folate receptor were used as control [36]. Atomic Absorption Spectroscopy (AAS) analysis reported in Figure 3A of cells incubated with Rho-FA_x_-AuNPs shows negligible cell association of the Rho-FA_x_-AuNPs with the MCF-7^FR-low^ cells with a significantly higher uptake by KB^FR-high^ cells increasing with the FA density and achieving a plateau for Rho-FA_50_-AuNPs, corresponding to 19,387 AuNPs/cell. This clearly confirms selective cell uptake is dependent of FR expression and FA on the NPs.

Cytofluorimetric analysis confirmed the association profiles obtained by the AAS analysis (Figure 3B) showing a progressively increased association of AuNP to KB cells as the FA density increased with respect to non-targeted Rho-FA_0_-AuNPs.

**Figure 4:**
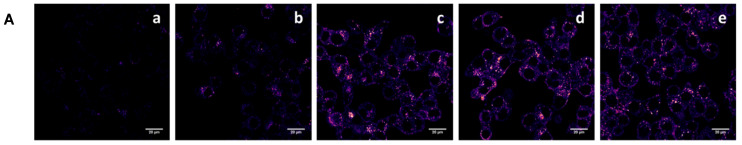

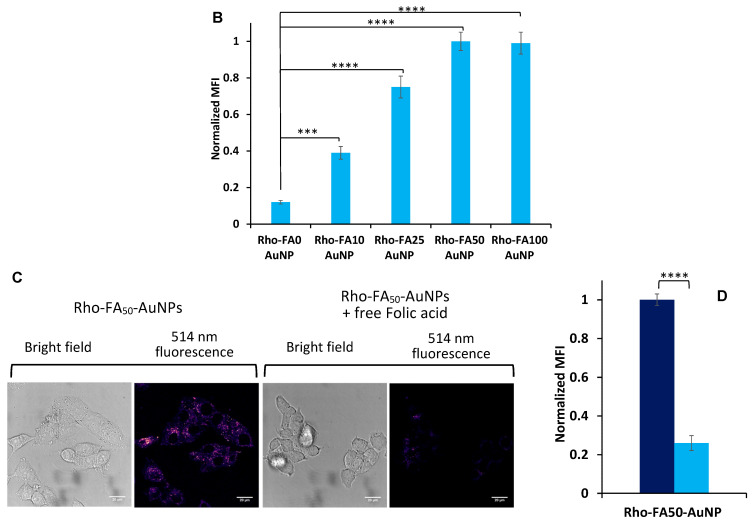
(**A**) Confocal microscopic images of KB^FR-high^ cells incubated with Rhodamine labelled gold nanoparticles decorated with increasing FA-PEG_3.5kDa_-SH units: 0, 10, 25, 50 and 100 units per particle (a, b, c, d, e, respectively). Scale bars: 20 µm. Magnification of white squared area of “d” is in Figure S3. (**B**) Normalized Mean Fluorescence Intensity of the confocal images of KB^FR-high^ cells incubated with Rho-FA_x_-AuNPs. Data were normalized to the Mean Fluorescence Intensity value of cells incubated with Rho-FA_50_-AuNPs and data are reported as the average ± SD of three independent experiments performed in triplicates. (**C**) Representative confocal microscopic and bright field images of KB^FR-high^ cells incubated with Rho-FA_50_-AuNPs in the presence or not of 200 µM free FA as competitive agent for FR binding. Scale bars: 20 µm. (**D**) Normalized Mean Fluorescence Intensity of confocal microscopic images of KB cells incubated with Rho-FA_50_-AuNPs in the presence (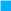) or not (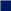) of free folic acid. The data were normalized to the Mean Fluorescence Intensity value of cells incubated with particles without folic acid. Data are reported as the mean ± SD of three independent experiments performed in triplicate. Statistical significance: *** p < 0.001, **** *p* < 0.0001.

The representative images of the confocal microscopy studies performed with KB^FR-high^ cells incubated with Rho-FA_x_-AuNPs in Figure 4A show increased particle internalization as the FA-PEG_3.5kDa_-SH density on the AuNPs surface increased. In all images (Figure 4A(b–e)) punctate labelling indicative of endolysosomes is prominent and there is little evidence of aggregation or significant accumulation on the plasma membrane (Figure S3 for cell magnification).

Quantitative analyses of the confocal images reported in Figure 4B show that the increase of FA density on AuNP surface from 0.015 to 0.037 FA-PEG_3.5kDa_-SH/nm^2^ results in a considerable increase of the cell association, which plateaus above 0.037 FA-PEG_3.5kDa_-SH/nm^2^.

Competitive studies were also performed to assess the cell association selectivity of FA-targeted nanoparticles. Figure 4C,D shows that the presence of 200 µM free FA in the competition experiment markedly inhibits the Rho-FA_50_-AuNP association to KB^FR-high^ cells.

### 3.5. Intracellular Trafficking Study

#### Lysosomal Disposition

The lysosomal compartment represents a destination of the active endocytic process where biological materials internalized from the extracellular space or delivered from intracellular locations e.g., during autophagy are degraded by a cocktail of enzymes [48,49]. Nanocarrier systems are also designed to release small molecule drugs from this location, including those targeting the FR with FA [50,51] Enhanced and sometimes unnatural trafficking to lysosomal compartments was observed for receptors when they were induced to cluster on the plasma membrane. This was reported for ErbB receptors [8,52], epidermal growth factor receptor, rabies G protein [53], transferrin receptor (TfR) [8,54] and also FR [55,56]. However, the correlation between the kinetic of this event and the degree of nanocarrier multivalency required to induce FR clustering has not been yet elucidated.

**Figure 5:**
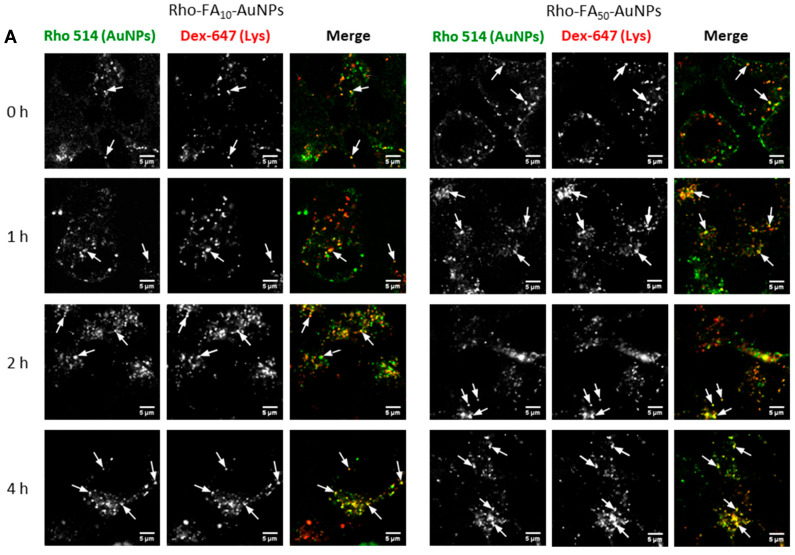

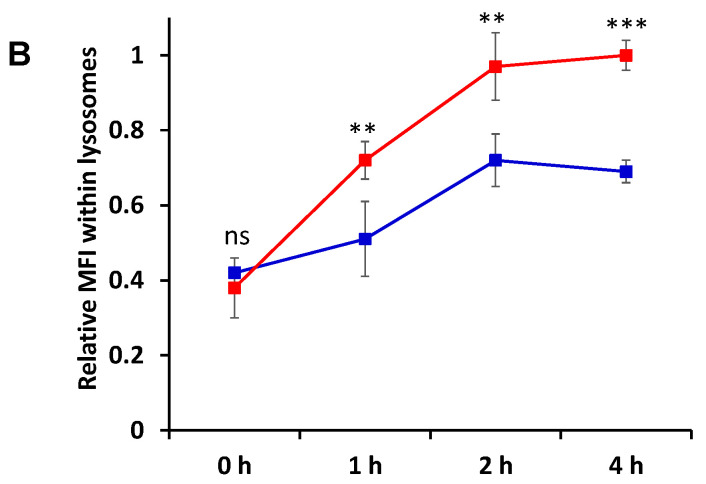
(**A**) Representative confocal microscopic images of KB^FR-high^ cells treated with Dex-647 to label lysosomes, and then pulsed-chased with Rho-FA_10_-AuNPs and Rho-FA_50_-AuNPs for 30 min at 37 °C in 5% CO_2_ atmosphere. Live cells were imaged at 0, 1, 2 and 4 h post-incubation. Arrows point to spots of Rho-FA_x_-AuNP colocalization within the lysosomes. Scale bars: 5 µm. (**B**) Relative Mean Fluorescence intensity of Rho-FA_10_-AuNPs (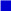) and Rho-FA_50_-AuNPs (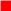) within lysosomal compartments of KB^FR-high^ cells over time. The data were normalized to the Mean Fluorescence intensity value of Rho-FA_50_-AuNPs at 4 h post-incubation. Error bars represent SD between mean normalized values of three independent experiments. Statistical analysis was performed for Rho-FA_10_-AuNPs vs Rho-FA_50_-AuNPs at each timepoint. Statistical significance: ns: *p* > 0.05, ** *p* < 0.01, *** *p* < 0.001.

The effect of AuNP surface folate density on the lysosomal disposition efficiency and rate was investigated by using low and high ligand surface density AuNPs, Rho-FA_10_-AuNPs and Rho-FA_50_-AuNPs, respectively, while FA-C_5_-Rho was used as reference. Cells were pulse-chased with the fluid-phase endocytosis probe Dex-647 to specifically label lysosomes [57] and imaged at scheduled times after incubation with targeted AuNPs for between 0 and 4 h. Incubation time with particles was selected according to the FR saturation time by folate conjugates reported in the literature [58]. The representative images of the KB^FR-high^ cells incubated with Rho-FA_10_-AuNPs and Rho-FA_50_-AuNPs reported in Figure 5A (Figure S4 for magnification of representative images) and the quantitative analysis of the confocal microscopic images reported in Figure 5B show time dependent increase in colocalization for both types of AuNPs with Dex-647 labelled lysosomes. After 30 min cell treatment, Rho-FA_10_-AuNPs and Rho-FA_50_-AuNPs were colocalized with Dex-647 with similar relative intensity indicating that their disposition into lysosomes takes place during the 30 min incubation, and reach the maximum accumulation in 2 h post-treatment. Since particles have the same amount of rhodamine (Table S1), the intensity of fluorescence found in the lysosomes correlates with the concentration of particles into these compartments. The cells treated with Rho-FA_10_-AuNPs display 30% lower MFI relative to lysosomes at 2 h post-treatment in comparison to cells incubated with Rho-FA_50_-AuNPs, indicating a lower particle accumulation in this compartment. This evidence supports our previous findings of higher Rho-FA_50_-AuNP association with KB^FR-high^ cells (Figure 4) with respect to Rho-FA_10_-AuNPs.

**Figure 6:**
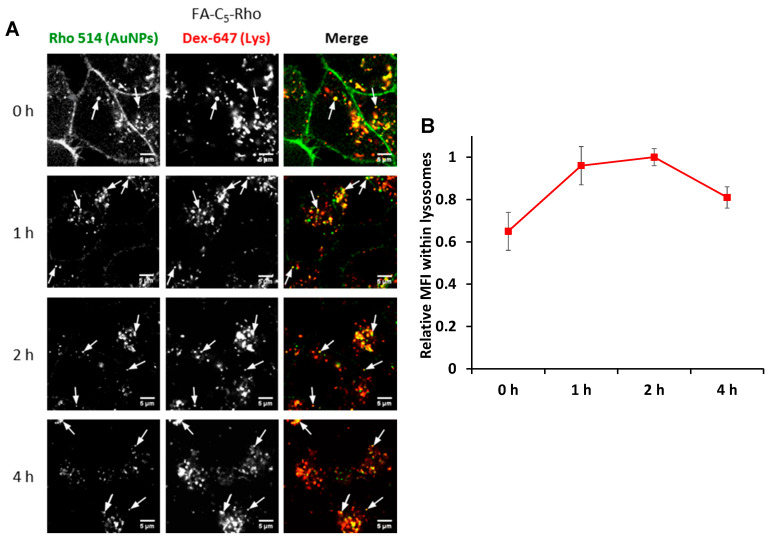
(**A**) Representative images of KB cells pre-incubated with Dex-647 for lysosomal labelling, and then incubated with monovalent FA-C_5_-Rho for 30 min. Live cells were imaged at 0, 1, 2 and 4 h post-treatment. Arrows point to spots of colocalization of FA-C_5_-Rho within the lysosomes. Scale bars: 5 µm. (**B**) Relative Mean Fluorescence intensity of FA-C_5_-Rho within lysosomal compartments of KB^FR-high^ cells at 0, 1, 2 and 4 h post-incubation. Data were normalized to the MFI value of FA-C_5_-Rho at 2 h. Error bars represent SD between mean normalized values of three independent experiments.

The lysosomal accumulation of FA-C_5_-Rho was investigated to get additional information about the effect of targeting agents on lysosomal disposition of AuNPs. The confocal images of KB^FR-high^ cells incubated with FA-C_5_-Rho reported in Figure 6A (Figure S4 for magnification of representative images) show that at the very earliest time point (designated time 0) the monovalent ligand is mostly located on the plasma membrane and most likely as a FA-C_5_-Rho-FR complex. Figure 6B shows that after 1 h incubation, there is considerable internalization of the ligand and that the FA-C_5_-Rho lysosomal accumulation reaches a plateau in 1–2 h. A slight decrease of the FA-C_5_-Rho content in the lysosomes was detected after 2 h post-incubation, which may be partially ascribable to the recycling of FR and release of FA-C_5_-Rho in the medium [58]. Overall, these results seem to indicate that the maximum FA-C_5_-Rho trafficking to lysosomes is achieved faster for the free ligand compared to the ligand decorated nanoparticles. The fate of FA-C_5_-Rho within lysosomes seems to diverge with respect to particles between 2 and 4 h, which could be due to differences in endocytic traffic, including recycling.

**Figure 7:**
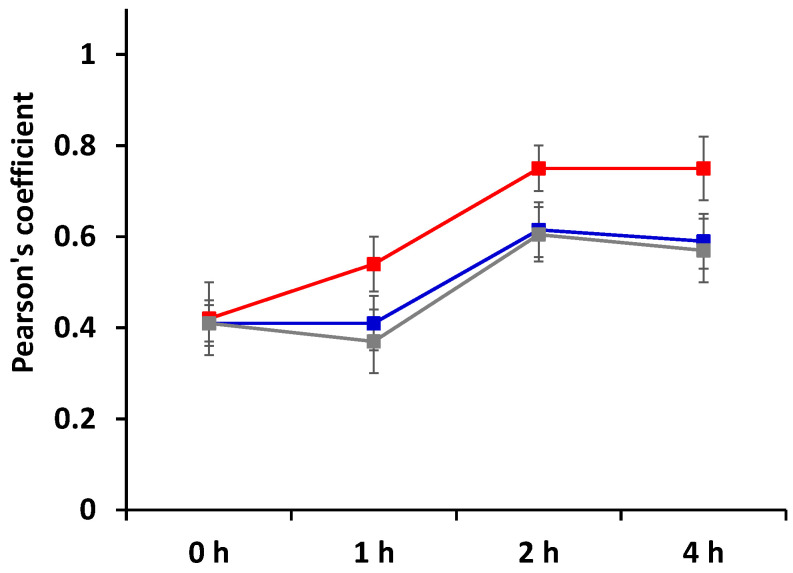
Pearson’s Coefficient indicating the colocalization in KB^FR-high^ cells of Rho-FA_10_-AuNPs (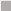), Rho-FA_50_-AuNPs (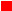) and the monovalent conjugate FA-C_5_-Rho (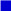) with Dex-647 used as probe for lysosomes. Statistical analysis is reported in Table S2.

Figure 7 reports the colocalization of either free FA or NP associated in lysosomes evaluated by Pearson’s coefficient (PC), which is calculated as the “r” value for the estimation of overlapping pixel of two channels of the same image. PC allows for comparing the lysosome disposition of fluorescent systems regardless the intracellular concentration and relative fluorescence intensity of the sample. Cells incubated with Rho-FA_50_-AuNPs showed a linear increase in PC between 0 and 2 h post-treatment, achieving a maximum value of 0.75 in 4 h, which corresponds to very high colocalization of the particles within the lysosomal compartments. AuNPs decorated with the lower FA density (Rho-FA_10_-AuNPs) reached a maximum PC value of 0.57 at 2 h post-treatment, resulting in a 24% lower colocalization with lysosomes than Rho-FA_50_-AuNPs. Of note, the profile of Rho-FA_10_-AuNPs almost overlaps with the one displayed by the monovalent FA-C_5_-Rho, hence prompting the hypothesis that the low ligand surface density is unable to induce receptor (FAR) clustering that modifies intracellular uptake and traffic and thus the behaviour of this NP is closer to that of the monovalent ligand.

Overall, the results of the Pearson’s coefficient deconvolution indicate that the higher the density of folate on the particle surface the faster and higher is the migration of particles to the lysosomes, which however, achieve a steady condition after approximately 2 h uptake. This is the first time that a comparative study derives the kinetic profile of lysosome access of multivalent nanoparticles with different folate density. We provide here insights concerning the effect of FA, and possibly other ligand, density on the kinetics of internalization and traffic of these targeting nanosystems in comparison with the soluble ligand that should follow the physiological profile.

**Figure 8:**
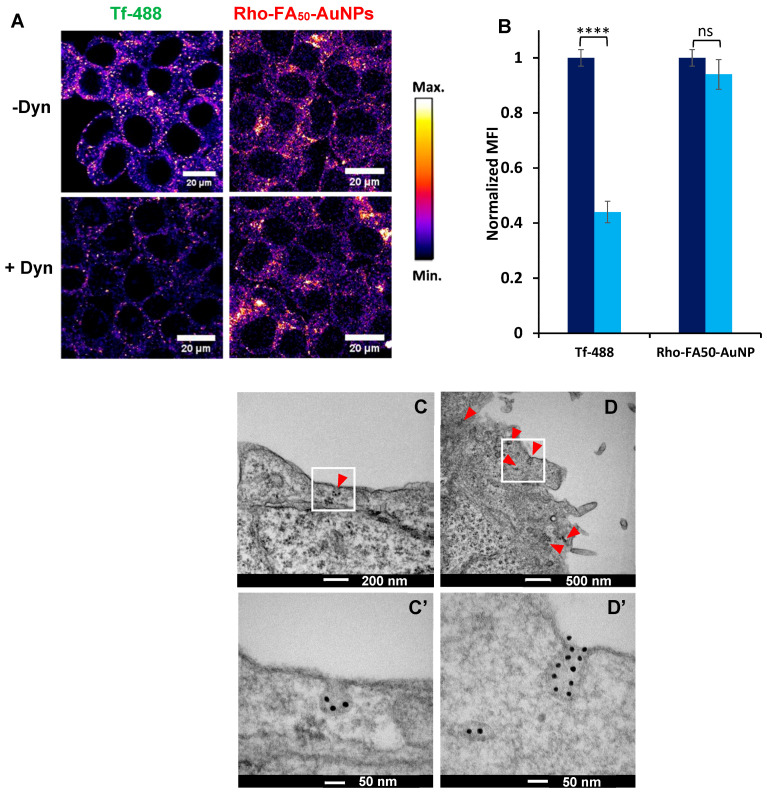
Rho-FA_50_-AuNP dynamin-independent cell uptake. (**A**) Confocal microscopy images of KB cells incubated with AlexaFluor488 labelled Transferrin (Tf-488—left panels), and Rho-FA_50_-AuNPs (right panels) in the presence (+ Dyn) or not (-Dyn) of Dynasore. (**B**) Relative Mean Fluorescence intensity of Tf-488 and Rho-FA_50_-AuNPs in the presence (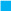) or not (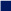) of Dynasore. The data were normalized to the Mean Fluorescence intensity value of cells non treated with Dynasore. Error bars represent SD between mean normalized values of three independent experiments. Statistical significance: ns: *p* > 0.05, **** *p* < 0.0001. (**C**–**D’**) TEM images of KB^FR-high^ cells incubated with Rho-FA_50_-AuNPs. Red arrows point to AuNPs. Panel (**C’**,**D’**) are magnifications of the white boxes of panel (**C**,**D**), respectively. Black dots are AuNP.

The evidence on cell uptake and intracellular disposition indicates that high folate density yields high intracellular delivery. To further investigate the gold nanoparticle trafficking to the lysosomes, KB^FR-high^ cells were pre-treated with dynasore, a non-competitive inhibitor of dynamin II, a GTPase protein also involved in the clathrin-dependent coated vesicle formation [59]. The cells were then incubated with Rho-FA_50_-AuNPs. AlexaFluor488 labelled Transferrin (Tf-488) was used as positive control for clathrin/dynamin-depended uptake [60]. The results described in Figure 8A,B show the inhibitory effects of dynasore on Tf-488 uptake whereas no noticeable effects were seen for the NPs. This suggests that cell uptake of Rho-FA_50_-AuNPs takes place via dynamin-independent mechanisms. Despite the limitations associated with using dynasore as a selective inhibitor of clathrin mediated endocytosis [61,62], the results are also in agreement with the data reported in the literature showing that the increase of folate density on 62–66 nm polystyrene nanoparticles shifts the cell uptake mechanism from predominantly clathrin- to predominantly caveolae-mediated [16]. On the other hand, another study reported by Dalal et al. [29] shows that the increase of folate density on quantum dots shifts the cell uptake mechanism from predominantly clathrin- to predominantly caveolae-mediated. It should be noted that while our system and the one by Moradi et al. folic acid is conjugated to the particles through a spatially low-constraint arm, in the case of Dalal et al. the particles were obtained by folate conjugation on the side chains of monomers and the resulting polymeric coating was rigidified by crosslinking. Therefore, the folate conjugated to this complex structure on particle surface may be at least partially buried in the shell and its mobility freedom to bind the cell FR can be limited. While it is difficult to correlate exposure and spatial freedom of the ligand to the particle uptake mechanism, our system and the one proposed by Moradi et al. ensures higher mobility to the ligand which may implies a different clustering profile of the receptor on cell membrane resulting in different uptake pathway.

TEM images of KB cells incubated with Rho-FA_50_-AuNPs generally support the results of fluorescence microscopy. Figure 8C,D shows Rho-FA_50_-AuNPs (black dots) in invaginations of different morphologies, including flask-shaped, indicative of caveolae.

These flask-shaped plasma membrane invaginations with a dimension of about 50–80 nm are typical of caveolae enriched structures that internalize material in a process often called potocytosis [63,64,65]. Quantitative analyses of the FR distribution on the plasma membrane have shown that administration of multivalent particles induces a substantial enrichment of FR clusters colocalized with caveolin in caveolae [11,22]. The size and shape of plasma membrane invaginations in the TEM images of Figure 8C’,D’ are in agreement with these studies. Clathrin coated pits appear on TEM studies like these as structures with a clear coat that are easy to see via this method [66] but were not visible in the NP-containing invaginations of KB cells in Figure 8C’,D’. However, the elucidation of the cell uptake mechanism requires a more sophisticated characterization.

Notably, the particles localize within these forming vesicles on cell membrane in a quite ordered pattern without aggregation suggesting that the PEG coating of the AuNPs prevents aggregation also when they get in contact within the cell membrane [67].

## 4. Conclusions

Our findings demonstrate that the density of targeting agents on the surface of nanoparticles does not affect only the cell interaction efficiency, but also the kinetic process of endolysosome disposition, which is crucial for drug delivery and its activity. Indeed, higher folate density results in higher and faster lysosomal disposition, which exposes the drug delivery system to a low-pH and enzyme-rich microenvironment. Therefore, high surface folate density may be a suitable strategy in the case of drug delivery systems that exploit lysosmal conditions to yield drug release. On the other hand, rapid lysosomal delivery may be detrimental if there is a need for endosomal escape of a lysosome fragile biological therapeutic, such as mRNA/siRNA peptide/protein. It should also be noted that the ‘endocytic profiles’ of cells has a major effect on the rate of traffic to lysosomes and also release of a cargo to the cytosol [68,69].

## Data Availability

The data presented in this study are available on request from the corresponding author.

## Supplementary Materials

The following are available online at https://www.mdpi.com/article/10.3390/pharmaceutics15030864/s1, Table S1: Composition of Rhodamine labelled Folate targeted AuNP formulations, size and PDI. Table S2: Statistical 2 way ANOVA analysis of colocalization in KB^FR-high^ cells of Rho-FA_10_-AuNPs, Rho-FA_50_-AuNPs and the monovalent conjugate FA-C_5_-Rho with lysosomes reported in Figure 7. Figure S1: MALDI-TOF spectra of NH_2_-PEG_3.5kDa_-SH (A) and FA-PEG_3.5kDa_-SH conjugate (B). Figure S2: MALDI TOF spectra of starting NH_2_-PEG_2kDa_-SH (A) and of Rho-PEG_2kDa_-SH conjugate (B). Figure S3: Magnification of the white squared area in Figure 5, panel d of confocal microscopic image of KB^FR-high^ cells incubated with Rho-FA_50_-AuNPs at pH 7.4. Scale bars: 20 µm. Figure S4. Magnification of confocal microscopic image of KB^FR-high^ cells treated with Dex-647 to label lysosomes, and then pulsed-chased with Rho-FA10-AuNPs (A) Rho-FA50-AuNPs (B) and FA-C5-Rho (C) for 30 minutes and imaged 4 h post-incubation. Scale bars: 5 µm.

## References

[1] Attia M.F., Anton N., Wallyn J., Omran Z., Vandamme T.F. (2019). An overview of active and passive targeting strategies to improve the nanocarriers efficiency to tumour sites. J. Pharm. Pharmacol..

[2] Rosenblum D., Joshi N., Tao W., Karp J.M., Peer D. (2018). Progress and challenges towards targeted delivery of cancer therapeutics. Nat. Commun..

[3] Kirpotin D.B., Drummond D.C., Shao Y., Shalaby M.R., Hong K., Nielsen U.B., Marks J.D., Benz C.C., Park J.W. (2006). Antibody targeting of long-circulating lipidic nanoparticles does not increase tumor localization but does increase internalization in animal models. Cancer Res..

[4] Austin C.D., De Mazière A.M., Pisacane P.I., van Dijk S.M., Eigenbrot C., Sliwkowski M.X., Klumperman J., Scheller R.H. (2004). Endocytosis and sorting of ErbB2 and the site of action of cancer therapeutics trastuzumab and geldanamycin. Mol. Biol. Cell.

[5] Ren X.-R., Wei J., Lei G., Wang J., Lu J., Xia W., Spector N., Barak L.S., Clay T.M., Osada T. (2012). Polyclonal HER2-specific antibodies induced by vaccination mediate receptor internalization and degradation in tumor cells. Breast Cancer Res..

[6] Hommelgaard A.M., Lerdrup M., van Deurs B. (2004). Association with membrane protrusions makes ErbB2 an internalization-resistant receptor. Mol. Biol. Cell.

[7] Longva K.E., Pedersen N.M., Haslekås C., Stang E., Madshus I.H. (2005). Herceptin-induced inhibition of ErbB2 signaling involves reduced phosphorylation of Akt but not endocytic down-regulation of ErbB2. Int. J. Cancer.

[8] Moody P.R., Sayers E.J., Magnusson J.P., Alexander C., Borri P., Watson P., Jones A.T. (2015). Receptor crosslinking: A general method to trigger internalization and lysosomal targeting of therapeutic receptor: Ligand complexes. Mol. Ther..

[9] Wymant J.M., Sayers E.J., Muir D., Jones A.T. (2020). Strategic Trastuzumab Mediated Crosslinking Driving Concomitant HER2 and HER3 Endocytosis and Degradation in Breast Cancer. J. Cancer.

[10] Zhu W., Okollie B., Artemov D. (2007). Controlled internalization of Her-2/neu receptors by cross-linking for targeted delivery. Cancer Biol. Ther..

[11] Mayor S., Rothberg K.G., Maxfield F.R. (1994). Sequestration of GPI-anchored proteins in caveolae triggered by cross-linking. Science.

[12] Montet X., Funovics M., Montet-Abou K., Weissleder R., Josephson L. (2006). Multivalent effects of RGD peptides obtained by nanoparticle display. J. Med. Chem..

[13] Elias D.R., Poloukhtine A., Popik V., Tsourkas A. (2013). Effect of ligand density, receptor density, and nanoparticle size on cell targeting. Nanomed. Nanotechnol. Biol. Med..

[14] Zhao M., Kircher M.F., Josephson L., Weissleder R. (2002). Differential conjugation of tat peptide to superparamagnetic nanoparticles and its effect on cellular uptake. Bioconj. Chem..

[15] Giljohann D.A., Seferos D.S., Patel P.C., Millstone J.E., Rosi N.L., Mirkin C.A. (2007). Oligonucleotide loading determines cellular uptake of DNA-modified gold nanoparticles. Nano Lett..

[16] Moradi E., Vllasaliu D., Garnett M., Falcone F., Stolnik S. (2012). Ligand density and clustering effects on endocytosis of folate modified nanoparticles. RSC Adv..

[17] Saha A., Basiruddin S.K., Maity A.R., Jana N.R. (2013). Synthesis of nanobioconjugates with a controlled average number of biomolecules between 1 and 100 per nanoparticle and observation of multivalency dependent interaction with proteins and cells. Langmuir.

[18] Woythe L., Tito N.B., Albertazzi L. (2021). A quantitative view on multivalent nanomedicine targeting. Adv. Drug Deliv. Rev..

[19] Sapsford K.E., Algar W.R., Berti L., Gemmill K.B., Casey B.J., Oh E., Stewart M.H., Medintz I.L. (2013). Functionalizing nanoparticles with biological molecules: Developing chemistries that facilitate nanotechnology. Chem. Rev..

[20] Rothberg K.G., Ying Y.S., Kolhouse J.F., Kamen B.A., Anderson R.G. (1990). The glycophospholipid-linked folate receptor internalizes folate without entering the clathrin-coated pit endocytic pathway. J. Cell Biol..

[21] Sabharanjak S., Sharma P., Parton R.G., Mayor S. (2002). GPI-anchored proteins are delivered to recycling endosomes via a distinct cdc42-regulated, clathrin-independent pinocytic pathway. Dev. Cell.

[22] Sabharanjak S., Mayor S. (2004). Folate receptor endocytosis and trafficking. Adv. Drug Deliv. Rev..

[23] Slowing I., Trewyn B.G., Lin V.S.Y. (2006). Effect of surface functionalization of MCM-41-type mesoporous silica nanoparticles on the endocytosis by human cancer cells. J. Am. Chem. Soc..

[24] Yang H., Lou C., Xu M., Wu C., Miyoshi H., Liu Y. (2011). Investigation of folate-conjugated fluorescent silica nanoparticles for targeting delivery to folate receptor-positive tumors and their internalization mechanism. Int. J. Nanomed..

[25] Niu L., Meng L., Lu Q. (2013). Folate-Conjugated PEG on single walled carbon nanotubes for targeting delivery of doxorubicin to cancer cells. Macromol. Biosci..

[26] Suen W.-L.L., Chau Y. (2014). Size-dependent internalisation of folate-decorated nanoparticles via the pathways of clathrin and caveolae-mediated endocytosis in ARPE-19 cells. J. Pharm. Pharmacol..

[27] Li Y.-L., Van Cuong N., Hsieh M.-F. (2014). Endocytosis pathways of the folate tethered star-shaped PEG-PCL micelles in cancer cell lines. Polymers.

[28] Cao D., Tian S., Huang H., Chen J., Pan S. (2015). Divalent folate modification on PEG: An effective strategy for improving the cellular uptake and targetability of PEGylated polyamidoamine–polyethylenimine copolymer. Mol. Pharm..

[29] Dalal C., Saha A., Jana N.R. (2016). Nanoparticle Multivalency Directed Shifting of Cellular Uptake Mechanism. J. Phys. Chem. C.

[30] Brazzale C., Mastrotto F., Moody P., Watson P.D., Balasso A., Malfanti A., Mantovani G., Caliceti P., Alexander C., Jones A.T. (2017). Control of targeting ligand display by pH-responsive polymers on gold nanoparticles mediates selective entry into cancer cells. Nanoscale.

[31] Sims G.E.C., Snape T.J. (1980). A method for the estimation of polyethylene glycol in plasma protein fractions. Anal. Biochem..

[32] Ellman G.L. (1959). Tissue sulfhvdrvl sroups. Arch. Biochem. Biophys..

[33] Turkevich J., Stevenson P.C., Hillier J. (1953). The formation of colloidal gold. J. Phys. Chem..

[34] Mastrotto F., Caliceti P., Amendola V., Bersani S., Magnusson J.P., Meneghetti M., Mantovani G., Alexander C., Salmaso S. (2011). Polymer control of ligand display on gold nanoparticles for multimodal switchable cell targeting. Chem. Commun..

[35] Liu X., Atwater M., Wang J., Huo Q. (2007). Extinction coefficient of gold nanoparticles with different sizes and different capping ligands. Colloids Surf. B Biointerfaces.

[36] Gallon E., Matini T., Sasso L., Mantovani G., Armiñan de Benito A., Sanchis J., Caliceti P., Alexander C., Vicent M.J., Salmaso S. (2015). Triblock copolymer nanovesicles for pH-responsive targeted delivery and controlled release of siRNA to cancer cells. Biomacromolecules.

[37] Roberts-Dalton H.D., Cocks A., Falcon-Perez J.M., Sayers E.J., Webber J.P., Watson P., Clayton A., Jones A.T. (2017). Fluorescence labelling of extracellular vesicles using a novel thiol-based strategy for quantitative analysis of cellular delivery and intracellular traffic. Nanoscale.

[38] Matini T., Francini N., Battocchio A., Spain S.G., Mantovani G., Vicent M.J., Sanchis J., Gallon E., Mastrotto F., Salmaso S. (2014). Synthesis and characterization of variable conformation pH responsive block co-polymers for nucleic acid delivery and targeted cell entry. Polym. Chem..

[39] Leamon C.P., Low P.S. (2005). Receptor-mediated drug delivery. Drug Delivery: Principles and Applications.

[40] Gabizon A., Horowitz A.T., Goren D., Tzemach D., Mandelbaum-Shavit F., Qazen M.M., Zalipsky S. (1999). Targeting folate receptor with folate linked to extremities of poly (ethylene glycol)-grafted liposomes: In vitro studies. Bioconj. Chem..

[41] Jain P.K., Lee K.S., El-Sayed I.H., El-Sayed M.A. (2006). Calculated absorption and scattering properties of gold nanoparticles of different size, shape, and composition: Applications in biological imaging and biomedicine. J. Phys. Chem. B.

[42] Link S., El-Sayed M.A. (1999). Spectral Properties and Relaxation Dynamics of Surface Plasmon Electronic Oscillations in Gold and Silver Nanodots and Nanorods.

[43] Park J.-W., Shumaker-Parry J.S. (2014). Structural study of citrate layers on gold nanoparticles: Role of intermolecular interactions in stabilizing nanoparticles. J. Am. Chem. Soc..

[44] Ulman A. (1996). Formation and structure of self-assembled monolayers. Chem. Rev..

[45] Bakshi A.K., Haider T., Tiwari R., Soni V. (2022). Critical parameters for design and development of multivalent nanoconstructs: Recent trends. Drug Deliv. Transl. Res..

[46] Wang J., Tian S., Petros R.A., Napier M.E., DeSimone J.M. (2010). The complex role of multivalency in nanoparticles targeting the transferrin receptor for cancer therapies. J. Am. Chem. Soc..

[47] Wang J., Min J., Eghtesadi S.A., Kane R.S., Chilkoti A. (2020). Quantitative study of the interaction of multivalent ligand-modified nanoparticles with breast cancer cells with tunable receptor density. ACS Nano.

[48] Schulze H., Kolter T., Sandhoff K. (2009). Principles of lysosomal membrane degradation: Cellular topology and biochemistry of lysosomal lipid degradation. Biochim. Biophys. Acta (BBA)-Mol. Cell Res..

[49] Ballabio A., Bonifacino J.S. (2020). Lysosomes as dynamic regulators of cell and organismal homeostasis. Nat. Rev. Mol. Cell Biol..

[50] Wang C., Wang Q., Wang H., Li Z., Chen J., Zhang Z., Zeng H., Yu X., Yang X., Yang X. (2023). Hydroxyethyl starch-folic acid conjugates stabilized theranostic nanoparticles for cancer therapy. J. Control. Release.

[51] Rana S., Shetake N.G., Barick K.C., Pandey B.N., Salunke H.G., Hassan P.A. (2016). Folic acid conjugated Fe_3_ O_4_ magnetic nanoparticles for targeted delivery of doxorubicin. Dalton Trans..

[52] Wang Q., Villeneuve G., Wang Z. (2005). Control of epidermal growth factor receptor endocytosis by receptor dimerization, rather than receptor kinase activation. EMBO Rep..

[53] Pierre C.A.S., Leonard D., Corvera S., Kurt-Jones E.A., Finberg R.W. (2011). Antibodies to cell surface proteins redirect intracellular trafficking pathways. Exp. Mol. Pathol..

[54] Liu A.P., Aguet F., Danuser G., Schmid S.L. (2010). Local clustering of transferrin receptors promotes clathrin-coated pit initiation. J. Cell Biol..

[55] Lee R.J., Low P.S. (1994). Delivery of liposomes into cultured KB cells via folate receptor-mediated endocytosis. J. Biol. Chem..

[56] Turek J.J., Leamon C.P., Low P.S. (1993). Endocytosis of folate-protein conjugates: Ultrastructural localization in KB cells. J. Cell Sci..

[57] Humphries Iv W.H., Szymanski C.J., Payne C.K. (2011). Endo-lysosomal vesicles positive for Rab7 and LAMP1 are terminal vesicles for the transport of dextran. PLoS ONE.

[58] Paulos C.M., Reddy J.A., Leamon C.P., Turk M.J., Low P.S. (2004). Ligand binding and kinetics of folate receptor recycling in vivo: Impact on receptor-mediated drug delivery. Mol. Pharmacol..

[59] Kirchhausen T., Macia E., Pelish H.E. (2008). Use of dynasore, the small molecule inhibitor of dynamin, in the regulation of endocytosis. Methods Enzymol..

[60] Zhai G., Wu J., Yu B., Guo C., Yang X., Lee R.J. (2010). A transferrin receptor-targeted liposomal formulation for docetaxel. J. Nanosci. Nanotechnol..

[61] Preta G., Cronin J.G., Sheldon I.M. (2015). Dynasore-not just a dynamin inhibitor. Cell Commun. Signal..

[62] Griffiths G., Gruenberg J., Marsh M., Wohlmann J., Jones A.T., Parton R.G. (2022). Nanoparticle entry into cells; the cell biology weak link. Adv. Drug Deliv. Rev..

[63] Anderson R.G.W. (1993). Potocytosis of small molecules and ions by caveolae. Trends Cell Biol..

[64] Conner S.D., Schmid S.L. (2003). Regulated portals of entry into the cell. Nature.

[65] Parton R.G., Del Pozo M.A., Vassilopoulos S., Nabi I.R., Le Lay S., Lundmark R., Kenworthy A.K., Camus A., Blouin C.M., Sessa W.C. (2020). Caveolae: The FAQs. Traffic.

[66] Tao-Cheng J.-H. (2020). Stimulation-induced differential redistributions of clathrin and clathrin-coated vesicles in axons compared to soma/dendrites. Mol. Brain.

[67] Suk J.S., Xu Q., Kim N., Hanes J., Ensign L.M. (2016). PEGylation as a strategy for improving nanoparticle-based drug and gene delivery. Adv. Drug Deliv. Rev..

[68] Vermeulen L.M.P., Brans T., Samal S.K., Dubruel P., Demeester J., De Smedt S.C., Remaut K., Braeckmans K. (2018). Endosomal size and membrane leakiness influence proton sponge-based rupture of endosomal vesicles. ACS Nano.

[69] Sayers E.J., Peel S.E., Schantz A., England R.M., Beano M., Bates S.M., Desai A.S., Puri S., Ashford M.B., Jones A.T. (2019). Endocytic profiling of cancer cell models reveals critical factors influencing LNP-mediated mRNA delivery and protein expression. Mol. Ther..

