# Supplementary material for: Influence of Folate-Targeted Gold Nanoparticles on Subcellular Localization and Distribution into Lysosomes
Source: Pharmaceutics. 2023 Mar 7;15(3):864. doi: 10.3390/pharmaceutics15030864 (PMC10053352; doi:10.3390/pharmaceutics15030864)
Supplement: Supplementary file 1 [file pharmaceutics-15-00864-s001.zip › pharmaceutics-2219256-supplementary.pdf]

Supplementary Information

Influence of folate-targeted gold nanoparticles on subcellular localization and distribution into lysosomes

Raffaella Daniele[1], Chiara Brazzale[1], Busra Arpac[1], Francesco Tognetti[1], Cristiano Pesce[1], Alessio Malfanti[2], Edward Sayers[3], Francesca Mastrotto[1], Arwyn T. Jones[3], Stefano Salmaso[1,*], Paolo Caliceti[1]

[1] Department of Pharmaceutical and Pharmacological Sciences, University of Padova, via F. Marzolo 5, 35131 Padova, Italy

[2] UCLouvain, Louvain Drug Research Institute, Advanced Drug Delivery and Biomaterials, Avenue Mounier 73 B1.73.12, 1200, Brussels, Belgium.

[3] Cardiff School of Pharmacy and Pharmaceutical Sciences, Cardiff University, Redwood Building, King Edward VII Ave, Cardiff CF10 3NB, United Kingdom.

* Correspondence: stefano.salmaso@unipd.it; Tel.: +390498271602

TABLES

Table S1. Composition of Rhodamine labelled Folate targeted AuNP formulations, size and PDI.

Sample Code	FA-PEG$_{3.5kDa}$-SH /AuNPs	Rho-PEG$_{2kDa}$-SH /AuNPs	Size (nm)	PDI
Rho-FA$_0$-AuNPs	0	500	28.6±5.4	0.28
Rho-FA$_{10}$-AuNPs	10	500	28.8±6.2	0.31
Rho-FA$_{25}$-AuNPs	25	500	29.7±5.8	0.33
Rho-FA$_{50}$-AuNPs	50	500	29.3±6.5	0.31
Rho-FA$_{100}$-AuNPs	100	500	29.1±6.1	0.28

Table S2. Statistical 2way ANOVA analysis of colocalization in KB$^{FR\text{-}high}$ cells of Rho-FA$_{10}$-AuNPs, Rho-FA$_{50}$-AuNPs and the monovalent conjugate FA-C$_5$-Rho with lysosomes reported in Figure 7.

time	Bonferroni's multiple comparisons test	Significant	Summary	Adjusted P Value
0 h				
	Rho-FA$_{10}$-AuNPs vs. Rho-FA$_{50}$-AuNPs	No	ns	>0.9999
	Rho-FA$_{10}$-AuNPs vs. Rho-C5-FA	No	ns	>0.9999
	Rho-FA$_{50}$-AuNPs vs. Rho-C5-FA	No	ns	>0.9999
1h				
	Rho-FA$_{10}$-AuNPs vs. Rho-FA$_{50}$-AuNPs	Yes	*	0.0493
	Rho-FA$_{10}$-AuNPs vs. Rho-C5-FA	NO	ns	>0.9999
	Rho-FA$_{50}$-AuNPs vs. Rho-C5-FA	Yes	**	0.0075
2 h				
	Rho-FA$_{10}$-AuNPs vs. Rho-FA$_{50}$-AuNPs	Yes	*	0.0393
	Rho-FA$_{10}$-AuNPs vs. Rho-C5-FA	No	ns	>0.9999
	Rho-FA$_{50}$-AuNPs vs. Rho-C5-FA	Yes	*	0.0248
4 h				
	Rho-FA$_{10}$-AuNPs vs. Rho-FA$_{50}$-AuNPs	Yes	*	0.0122
	Rho-FA$_{10}$-AuNPs vs. Rho-C5-FA	No	ns	>0.9999
	Rho-FA$_{50}$-AuNPs vs. Rho-C5-FA	Yes	**	0.0046

RESULTS

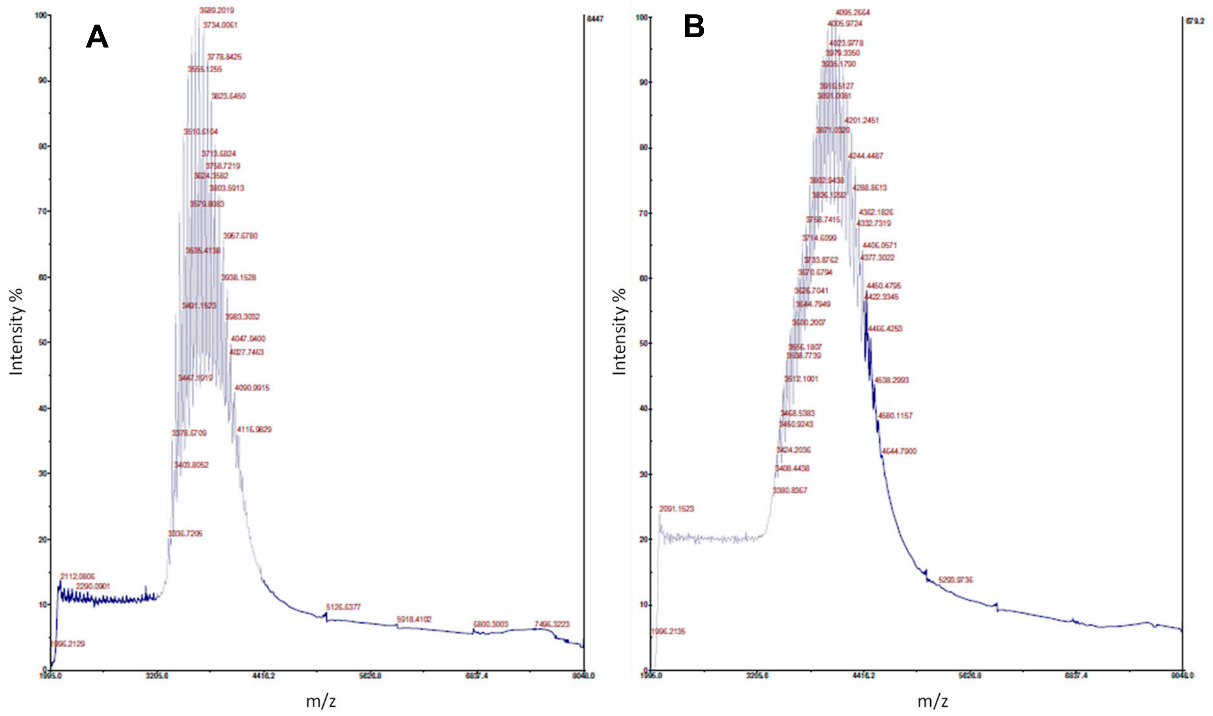

Figure S1. MALDI-TOF spectra of NH_2-$PEG_{3.5kDa}$-SH (A) and FA-$PEG_{3.5kDa}$-SH conjugate (B).

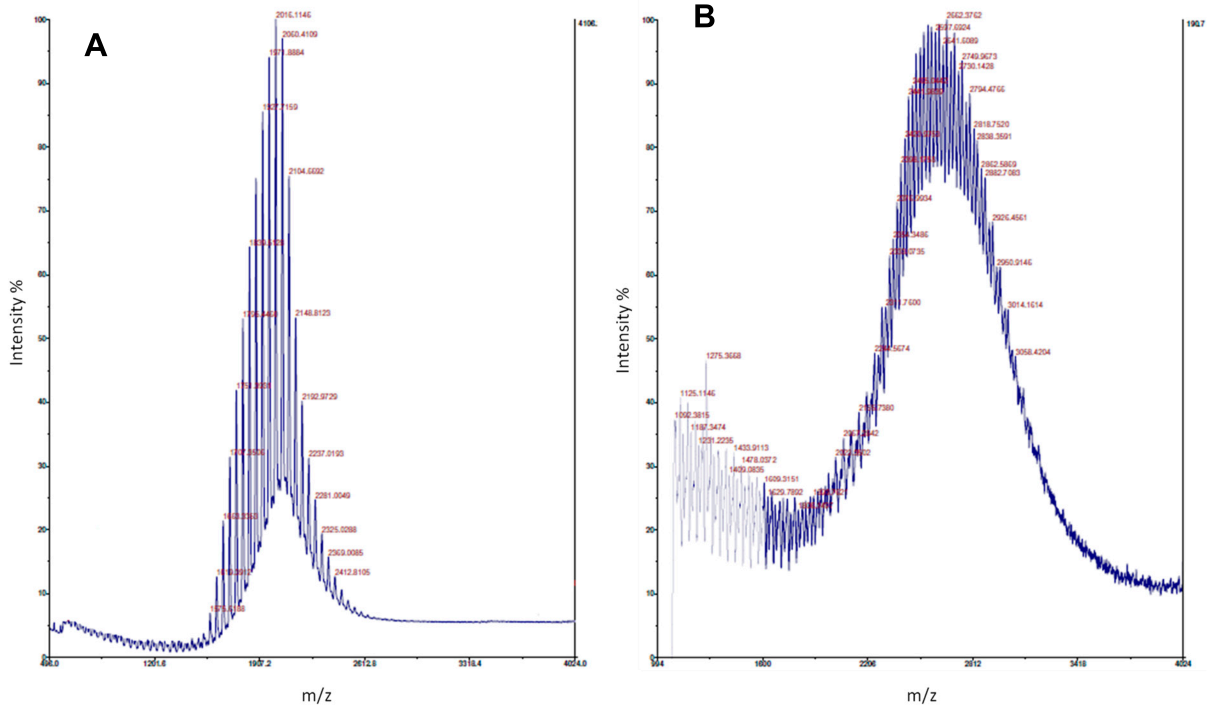

Figure S2. MALDI TOF spectra of starting NH_2-PEG_{2kDa}-SH (A) and of Rho-PEG_{2kDa}-SH conjugate (B).

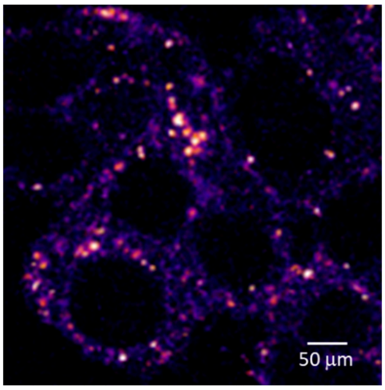

Figure S3. Magnification of the white squared area in Figure 5, panel d of confocal microscopic image of KB$^{FR\text{-}high}$ cells incubated with Rho-FA$_{50}$-AuNPs at pH 7.4. Scale bars: 20 µm.

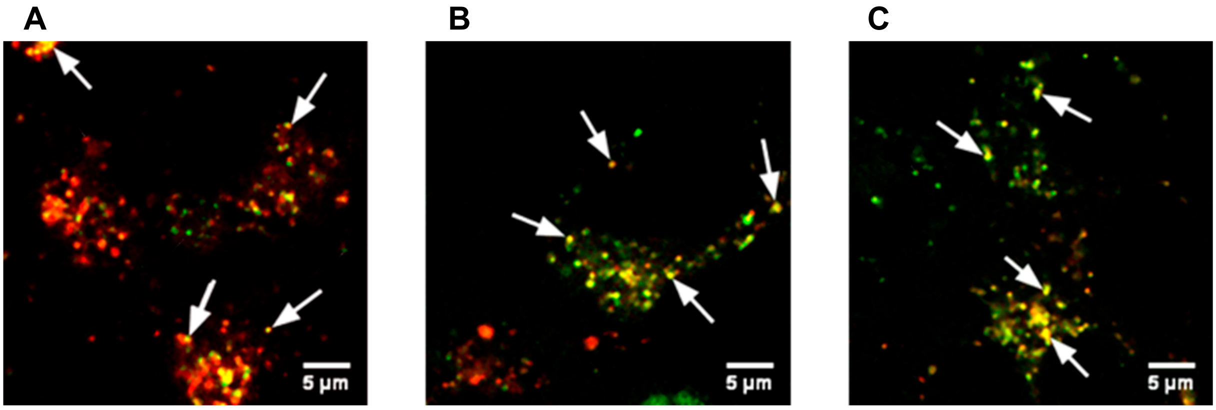

Figure S4. Magnification of confocal microscopic image of KB$^{FR\text{-}high}$ cells treated with Dex-647 to label lysosomes, and then pulsed-chased with Rho-FA$_{10}$-AuNPs (A) Rho-FA$_{50}$-AuNPs (B) and FA-C$_5$-Rho (C) for 30 minutes and imaged 4 h post-incubation. Scale bars: 5 µm.
